# A Wireless, High-Quality, Soft and Portable Wrist-Worn System for sEMG Signal Detection

**DOI:** 10.3390/mi14051085

**Published:** 2023-05-21

**Authors:** Zekai Liang, Xuanqi Wang, Jun Guo, Yuanming Ye, Haoyang Zhang, Liang Xie, Kai Tao, Wen Zeng, Erwei Yin, Bowen Ji

**Affiliations:** 1 Unmanned System Research Institute, Northwestern Polytechnical University, Xi’an 710072, China; liangzekai@mail.nwpu.edu.cn (Z.L.); xuanqi@mail.nwpu.edu.cn (X.W.); gj18831723531@163.com (J.G.); 2 Ministry of Education Key Laboratory of Micro and Nano Systems for Aerospace, School of Mechanical Engineering, Northwestern Polytechnical University, Xi’an 710072, China; taokai@nwpu.edu.cn (K.T.); zengwen@nwpu.edu.cn (W.Z.); 3 Innovation Center NPU Chongqing, Northwestern Polytechnical University, Chongqing 400000, China; 4 Queen Mary University of London Engineering School, Northwestern Polytechnical University, Xi’an 710072, China; ym.ye@mail.nwpu.edu.cn; 5 Defense Innovation Institute, Academy of Military Sciences (AMS), Beijing 100071, China; haoyang@tju.edu.cn (H.Z.); xielnudt@gmail.com (L.X.); 6 Tianjin Artificial Intelligence Innovation Center (TAIIC), Tianjin 300450, China

**Keywords:** wrist-worn system, soft electronic wristband, sEMG, muscle fatigue detection, gesture recognition

## Abstract

The study of wearable systems based on surface electromyography (sEMG) signals has attracted widespread attention and plays an important role in human–computer interaction, physiological state monitoring, and other fields. Traditional sEMG signal acquisition systems are primarily targeted at body parts that are not in line with daily wearing habits, such as the arms, legs, and face. In addition, some systems rely on wired connections, which impacts their flexibility and user-friendliness. This paper presents a novel wrist-worn system with four sEMG acquisition channels and a high common-mode rejection ratio (CMRR) greater than 120 dB. The circuit has an overall gain of 2492 V/V and a bandwidth of 15~500 Hz. It is fabricated using flexible circuit technologies and is encapsulated in a soft skin-friendly silicone gel. The system acquires sEMG signals at a sampling rate of over 2000 Hz with a 16-bit resolution and transmits data to a smart device via low-power Bluetooth. Muscle fatigue detection and four-class gesture recognition experiments (accuracy greater than 95%) were conducted to validate its practicality. The system has potential applications in natural and intuitive human–computer interaction and physiological state monitoring.

## 1. Introduction

In recent years, the development of sEMG detection systems has been propelled by advances in sensor technology, signal processing, and machine learning. These systems typically involve the use of electromyography electrodes to measure muscle activity in various body parts such as the arm [1,2], leg [3,4], and face [5], which are then amplified, filtered, and digitized for analysis. However, the performance of these systems is often limited by factors such as noise and the complexity of the signal itself [6]. Therefore, researchers have attempted to improve the accuracy of sEMG detection through various methods.

In terms of circuitry, high-pass and low-pass filters can be designed to filter out noise and interference signals, along with a notch filter to eliminate industrial frequency signal’s interference. For example, Strzecha et al. introduced a high-pass filter with a cutoff frequency of 2 Hz to eliminate direct current offset and low-frequency noise, resulting in improved signal quality [7]. Regarding algorithms, the time-domain period of interest can be windowed before signal classification to exclude confounding effects from other periods [8]. For instance, C. Tepe et al. achieved a classification accuracy of 95.8% for five fingers and one static gesture control of a prosthetic using preprocessed EMG signals with a 100 ms 50% overlapping window [9]. Regarding structural design, Prof. Li et al. designed the high-performance flexible microneedle array to pierce the outermost layer of the skin to directly acquire stable, low-resistance signals [10]. In terms of materials, the use of smart fabrics and metamaterial texts can effectively improve the sensing performance and comfort of wearable systems [11,12,13].

Meanwhile, research on the use of sEMG signals for gesture recognition [14,15,16] and physiological health status monitoring [17,18,19,20] has gained increasing attention in the fields of human–computer interaction and biomedical engineering. Prof. Sheng et al. developed a real-time gesture recognition wristband that combined sEMG and inertial measurement unit sensors located at the wrist instead of at the forearm, which was more in line with human-wearing habits. This system could identify eight airborne gestures and four surface gestures with accuracies of 92.6% and 88.8%, respectively. Prof. Chen et al. proposed a novel regression-based framework for the quantitative assessment of muscle spasticity using wearable sEMG and inertial sensors combined [19]. Prof. Liu et al. proposed a novel in-home sleep monitoring system with an eight-channel biopotential acquisition front-end chip, which could acquire sEMG signals and combine them with electrooculography and ECG signals for sleep state monitoring [20].

However, designing and implementing wearable surface sEMG sensor systems still encounter several challenges, including the need for high-quality signal acquisition and user comfort and convenience of sensor placement. In this study, we design and implement a novel gesture recognition system based on sEMG signals, which uses a wearable wristband sensor to capture muscle activity at the wrist and has been validated for practical applications.

Notably, two novelties of this article are highlighted as follows:

Firstly, high-quality signal acquisition. We designed a weak wrist muscle signal acquisition system characterized by high-gain, high-sampling frequency, and wide-bandwidth to capture more muscle signal features, which improves the accuracy of muscle signal decoding [21]. Additionally, we placed the signal acquisition circuit on the watch strap, reducing noise introduced by the circuitry [22]. The electrode points are in close contact with the skin by tightening the wristband, which improves the signal quality and effectively reduces the size of the core board circuit.

Secondly, comfort and convenience. We applied flexible circuits and flexible skin-friendly silicone to package the circuits, reducing the weight of the system while improving comfort. Compared with other muscle signal acquisition systems in different locations, the wristband system is more consistent with daily wearing habits.

## 2. System Design

### 2.1. General Architecture

**Figure 1:**
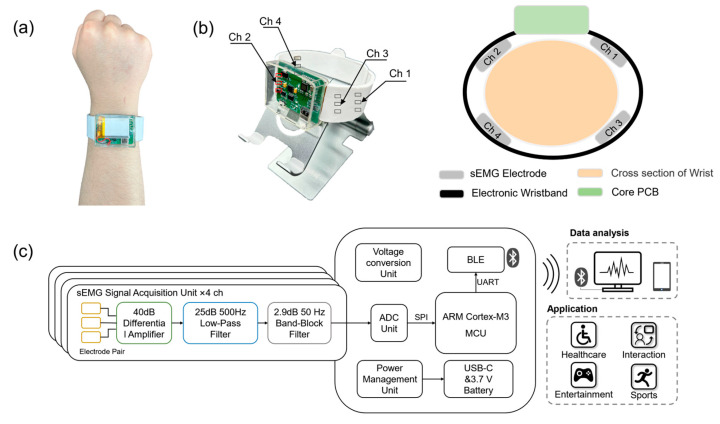
General design of the wrist-worn system. (**a**) Wearing method and position. (**b**) System appearance and sEMG electrode channel location (Ch 1~Ch 4 indicate Channel 1~4). (**c**) Overall system design architecture and application scenarios.

**Figure 2:**
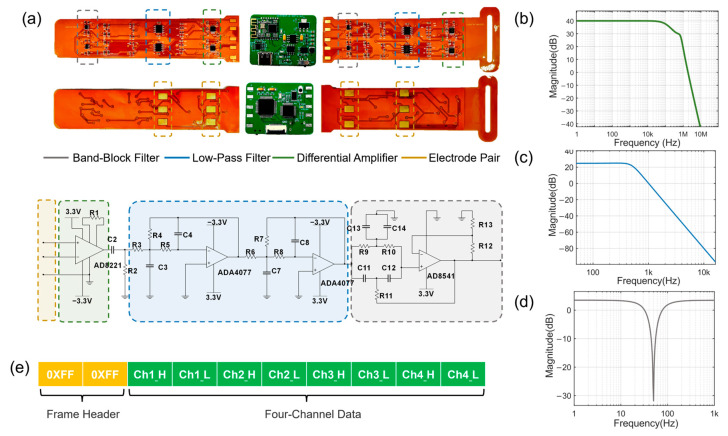
Design of the soft electronic wristband. (**a**) The overall circuit design composition and appearance. (**b**) The logarithmic amplitude and frequency characteristic curve of the differential amplifier circuit. (**c**) The logarithmic amplitude frequency characteristic curve of low-pass filter circuit. (**d**) The logarithmic amplitude frequency characteristic curve of the trap circuit. (**e**) Signal transmission data frame format.

The overall architecture of the developed wearable sEMG detection system for the wrist is illustrated in Figure 1. The system can be worn on the wrist (as shown in Figure 1a), with four-channel sEMG electrodes distributed on the inner side of the wristband, and the electrode points being in close contact with the skin on the wrist (as shown in Figure 1b). From a physical perspective, the system can be divided into the electronic wristband and the core board (as shown in Figure 1c and Figure 2a). The signal conditioning circuit on the electronic wristband is used to amplify and filter the signals. The controller and data processing circuit on the central core board perform analog-to-digital conversion of the signals and wirelessly transmit the data to a computer, smartphone, or other intelligent terminals for algorithm analysis. Ultimately, the wristband system achieves human–machine interaction, physiological health monitoring, and gesture semantic decoding applications.

### 2.2. Skin-Attached Electronic Wristband and System Package

We combined the flexible printed circuit board (FPCB) technology with flexible silicone material to design a skin-adhesive electronic wristband for sEMG signal conditioning. This design effectively improves the wearing comfort and reduces the size of the core circuit board. The FPCB was made of polyimide substrate (JLC Electronics Co., Ltd., Shenzhen, China). The manufacturing process of FPCB mainly includes the following steps:

(1) Substrate Preparation: The process begins with preparing the substrate material, which uses a flexible polymer membrane material: polyimide. The substrate is cleaned and coated with a layer of adhesive material to enhance adhesion;
(2) Photolithography: A photosensitive material called a photoresist is applied onto the substrate. The circuit design is then transferred onto the photoresist using photolithography techniques. Ultraviolet (UV) light is used to expose the photoresist through a photomask, creating a pattern that matches the circuit layout.
(3) Etching: After the photoresist is exposed, the unexposed areas are dissolved, leaving behind the desired circuit pattern on the substrate. Chemical etching is commonly used to selectively remove the unwanted copper or other conductive material.
(4) Plating: The exposed conductive areas are plated with additional layers of metal, typically copper, to increase the thickness and improve conductivity. This step helps reinforce the conductive traces and pads.
(5) Solder Mask Application: A solder mask layer is applied to the FPCB, except for the areas where the electrical connections need to be made. The solder mask protects the circuit from environmental factors and prevents unintentional short circuits.
(6) Component Attachment: Surface mount technology (SMT) or through-hole technology (THT) is used to attach electronic components onto the FPCB. SMT involves placing components onto solder pads and reflowing the solder to establish electrical connections. THT involves inserting components through drilled holes and soldering them on the opposite side.

The outer layer was encapsulated with a flexible and skin-friendly AB silicone mixture (SJ3220, SANJING Co., Ltd., Beijing, China) cured at room temperature to reduce internal stress and protect the circuit components. The cured silicone packaging material used has a Shore Hardness of 00-45, a Tensile Strength of 315 psi (Pounds per square inch), and an Elongation Break of 980%.

Then, a laser cutting machine (LR-QZ100T, Langrui Laser Technology Co., Ltd., Xi’an, China) was applied to process the silicone encapsulation’s outer contour. The sEMG electrodes were designed with a differential electrode pair, where three points were considered as one group: the upper and lower electrode points were differential electrodes, and the middle electrode point was a reference electrode. The electrode pair’s distance was designed to be 3.5 cm, and the electrode material was copper-plated nickel with good conductivity, with a size of 5 mm × 4 mm. The electrode wristband and the core circuit board were connected using soldering pads. The core circuit board and battery were encapsulated and protected by a 3D-printed shell made of transparent photosensitive resin.

### 2.3. Circuits and Electronics Unit Design

As shown in Figure 1c, from a functional perspective, the system circuit consists primarily of four modules: (1) sEMG Signal Acquisition Unit, (2) Control Unit, (3) Wireless Communication Unit, and (4) Power Management Unit.

#### 2.3.1. sEMG Signal Acquisition Unit

The sEMG signal acquisition unit can conduct amplification, filtering, and sampling of four channels of sEMG signals. The peak-to-peak value of sEMG signals on the human body is 0~6 mV [23], and the frequency is mainly distributed between 30 and 300 Hz [24]. The design of the wearable sEMG acquisition circuit for the wrist mainly considers the following requirements: (1) the amplitude of the wrist sEMG signal is smaller [16] and requires higher signal gain; (2) the signal is susceptible to DC offset, motion artifact, and power frequency interference [25], and therefore requires bandpass and bandstop filtering, with the bandwidth designed to cover the main frequency range of sEMG signals; (3) the sampling frequency of the signal should satisfy the Nyquist sampling theorem [26]. At the same time, in order to meet the requirements of time-domain feature analysis of muscle sEMG signals, the sampling frequency should be set to 4~5 times the frequency of the principal component signal [27].

Based on these requirements, as shown in Figure 2a, a three-stage amplification and filtering circuit was designed as a suitable solution. The first stage of amplification used a precision instrumentation amplifier chip (AD8221, Analog Devices, Inc., Wilmington, MA, USA) for differential amplification, and the gain was set to 100 V/V by setting the external resistor $R_{1}=\;499\;\Omega$ (1% accuracy), which ensured good amplification characteristics below 10 kHz. The logarithmic amplitude–frequency characteristic curve is shown in Figure 2b. Moreover, AD8221 has excellent AC characteristics, with a minimum CMRR of 120 dB (dc-60 Hz, G = 100) and a maximum voltage input noise of $8nV/\sqrt{Hz}$ (1 kHz).

Considering the impact of low-frequency motion artifacts and other interference signals on the subsequent amplification circuits, we employed a passive high-pass filter with a cutoff frequency of 15 Hz after the first-stage amplifier. For the design of the second-stage amplification and filtering circuit, we compared the response characteristics of Chebyshev, Butterworth, and Bessel filters [28] and selected the Butterworth filter featured by a good balance between the number of devices and frequency response characteristics. The system utilized a second-order fourth-order Butterworth low-pass filter with a cutoff frequency of 500 Hz (−3 dB) and a gain of 17.8 V/V. Additionally, the −40 dB stopband starting frequency was designed to be 1.6 kHz. The logarithmic amplitude–frequency characteristic curve is shown in Figure 2c. The chip selected for this circuit was the ADA4077-2 (Analog Devices, Inc.), which is a single-chip dual-channel amplifier with a wide bandwidth of 4 MHz, low noise of $7\;nV/\sqrt{Hz}$ (typical), high CMRR, and power supply rejection ratio (PSRR) (minimum values greater than 120 dB) to further reduce the number of components. All of these properties fully meet the requirements of the design of the second-stage amplification and filtering circuit.

The third-stage amplification and filtering circuit used a typical active twin-T notch filter (AD8541, Analog Devices, Inc.) to filter out power frequency noise. The equivalent quality factor $Q=\frac{1}{2(2-G)}$, and the gain $G=1+\frac{R_{b}}{R_{a}}$. Choosing $R_{b}=1\;k\Omega$ and $R_{a}=400\;\Omega$, and the gain was then designed to be 1.4 V/V. The logarithmic frequency response curve is shown in Figure 2d. Notably, it had a signal attenuation of below −30 dB near the 50 Hz power frequency signal. Finally, the amplification factor of the signal conditioning unit in the passband was 100×17.8×1.4=2492.

After circuit conditioning, the sEMG signal remains analog and cannot be directly processed using digital processors. Therefore, analog-to-digital conversion is necessary. Since most microcontroller unit (MCU) chips only sample positive voltages, an external analog-to-digital converter (ADC) chip (AD7606, Analog Devices, Inc.) was used for analog-to-digital conversion. The AD7606 is a true bipolar 16-bit charge-redistribution successive-approximation type ADC, with an analog input impedance of 1 MΩ. Additionally, it internally installs the analog input clamp protection, a second-order anti-aliasing filter, and a track-and-hold amplifier. It also has a high-speed serial and parallel interface, capable of synchronously sampling up to 8 channels at a maximum rate of 200 ksps. Considering the practical requirements for data throughput and power consumption, the sampling frequency was designed to be 2 kHz, while retaining the ability to increase the sampling frequency by changing the code. The sEMG Signal Acquisition Unit was designed to achieve high-quality signal conditioning and sampling, playing a key role in accurately processing and analyzing signals.

#### 2.3.2. Control Unit

The main function of the control unit is to control ADC sampling and data transfer among various units through an embedded program. The sampled data from the ADC was transferred to the MCU’s internal memory through the serial peripheral interface (SPI) protocol and then transmitted to the output buffer register of the Universal Asynchronous Receiver-Transmitter (UART) serial port through DMA operation. Finally, the data were transmitted through the wireless communication unit connected to the UART serial port. The controller used an ARM Cortex M3-based chip (STM32F103, STMicroelectronics, Geneva, Switzerland), which has a maximum operating frequency of 72 MHz, 2 channels of 12-bit ADC, 37 GPIO interfaces, 64 kb Flash, 2.6~3.6 V operating voltages, and supports communication protocols such as SPI, USART, and IIC, which is reasonable for the system design.

#### 2.3.3. Wireless Communication Unit

The wireless communication unit utilized BLE transmission technology, which has lower power consumption compared to Wi-Fi. Considering factors such as data throughput, real-time performance, and the ability of most mobile devices to support, lower power consumption Zigbee and NFC communication technologies were not selected. The RF chip selected was the HC-04S chip (HC-04S, HC TECH, Guangzhou, China), which supports SPP&BLE 5.0 communication protocols and can be easily connected to most wireless terminal products. The wireless working frequency band is 2.4 GHz ISM, and the modulation method is GFSK. The module’s maximum transmission power is 6 dBm, and the receiver sensitivity is −92 dBm. Through the design of the wireless communication unit, wireless transmission of data to smart terminals can be achieved, which provides higher flexibility compared to wired data transmission.

#### 2.3.4. Power Management and Voltage Conversion Units

The main function of the power management unit is to provide stable power supply to other units and to manage the battery charging process. Our wristband system was powered by a 600 mAh 3.7 V lithium polymer battery. The power supply design for the system is required as follows: (1) the amplifier chip needs a ±3.3 V power supply; (2) the MCU obtains a 3.3 V power supply; (3) the ADC acquisition chip requires a 5 V power supply. To meet these requirements, the following were designed: the 5 V power supply voltage was obtained through the DC-DC converter chip TPS63020 (TI, Dallas, TX, USA), which has an efficiency of over 90% and works at a fixed frequency of 2.4 MHz. The maximum output current can reach 3 A. The 3.3 V power supply voltage was converted by the 5–3.3 V LDO chip TLV1117LV (TI, Dallas, TX, USA), with a typical accuracy of 1.5% and a maximum output current of 1A. Because the LDO chip used requires a voltage difference of more than 1.5 V between the input voltage and output voltage, the voltage conversion scheme of “3.7 V–5 V–3.3 V” was chosen. The −3.3 V power supply voltage was obtained through the charge pump chip TL7660 (TI, Dallas, TX, USA, with a typical power efficiency of 98%. In addition, the system charged the battery through a standard USB Type-C interface and the power management chip TP4056 (UMW, Shenzhen, China). Due to its special internal MOSFET architecture and anti-reverse charging circuit, TP4056 does not require external detection resistors and isolation diodes. Its maximum charging current can reach 1000 mA, but considering charging time and safety, the charging current was designed to be 300 mA. By providing reliable and efficient power supply to all parts of the system, the power management unit can effectively improve the stability of the system.

### 2.4. Data Processing and Visualization

The sEMG data acquired by the wristband system are transmitted wirelessly via Bluetooth Low Energy (BLE) to the terminal for data processing and visualization. In the process of data transmission and conversion, the ADC firstly quantified the collected signal, output it as a binary two’s complement code, and transmitted the single-channel 16-bit hexadecimal data via the serial interface. The data were then stored in the microcontroller unit’s internal memory via the SPI bus that connected the ADC and the MCU. Subsequently, the MCU packaged the data in the format of a data frame shown in Figure 2e, controlled the UART serial port to transmit the data to the BLE module, and wirelessly transmitted it to the terminal. Finally, data were unpacked and analyzed on the terminal. The embedded program in the MCU was written in C language, and the real-time data visualization software for data waveforms was written using the LabView software. The data analysis and decoding algorithm was implemented using MATLAB and Python.

## 3. Results and Discussion

### 3.1. System Characterization

#### 3.1.1. sEMG Signal Acquisition Unit

**Figure 3:**
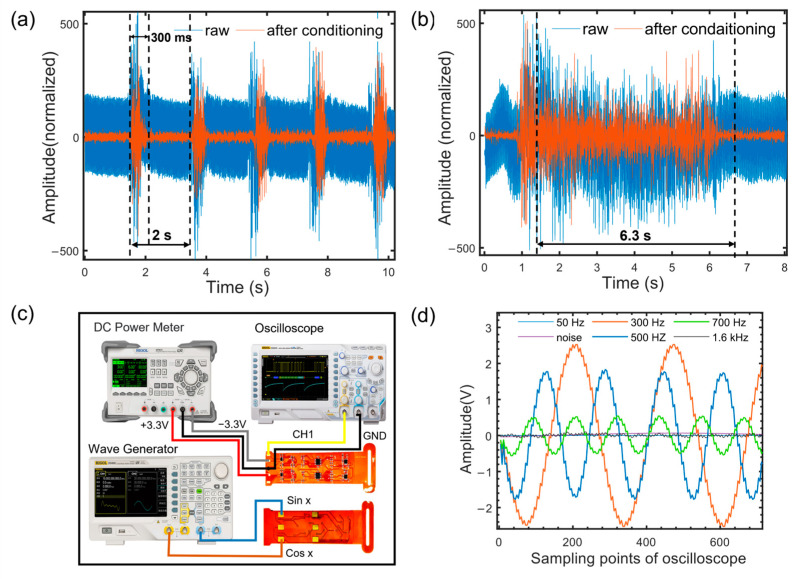
System performance characterization. (**a**) Raw signal versus system conditioning signal under intermittent firing conditions. (**b**) Raw signal versus system conditioning signal under continuous force conditions. (**c**) The measurement of the system acquisition channel gain. (**d**) System output gain voltage curves under different frequency output signal conditions.

We compared the sEMG signals acquired by our wristband system with those obtained from a commercial sEMG acquisition device (NeuSen WM, Neuracle Technology Co., Ltd., Changzhou, China). The positions and experimental protocols were the same for both devices. Figure 3a shows the periodic intermittent force-generated sEMG signals with a period of 2 s and force duration of 300 ms. Figure 3b shows the sustained force-generated sEMG signals with a duration of 6 s, with an actual detection time of 6.3 s. The comparative analysis of the two experiments indicated that, in comparison with the commercial sEMG acquisition device, our wristband system reduced the noise amplitude of the sEMG signals collected during periodic intermittent force and sustained force by 87.01% and 88%, respectively. The baseline drift of the sEMG signals was effectively suppressed.

We input different frequency $2mV_{pp}$ sine wave differential signals into the sEMG signal acquisition unit and calculated the ratio of input and output peak-to-peak amplitudes to measure the gain of each channel. The measurement process, as shown in Figure 3c, involved an external regulated DC power supply (DP831, RIGOL Technologies Co., Ltd., Suzhou, China) supplying power, a waveform generator (DG4062, RIGOL Technologies Co., Ltd., Suzhou, China) generating two channels of $2mV_{pp}$ sine wave signals, and one of the channels starting with a phase of 180° connected to the input terminal of the circuit for differential signal acquisition. We changed the input signal frequency, detected the amplitude and frequency of the output signal using an oscilloscope (DS2202A, RIGOL Technologies Co., Ltd., Suzhou, China) for 30 s, and analyzed the output signal after five measurements. Figure 3d shows the results of one of the measurements. The sEMG signal acquisition unit achieved a gain of 2488±3 V/V(67.92 dB) at 300 Hz and a gain of 1771±2 V/V(64.96 dB) at 500 Hz, with a decay ratio of 2.96 dB at the cutoff frequency. In the high-frequency range, the gain was 413±3 V/V at 700 Hz, 16±3 V/V at 1.6 kHz, and 43±2 V/V at 50 Hz. By shorting the two differential input channels, the equivalent input noise of the channels was measured to be 213±5 μV. The test results showed that our wristband system achieved good performance in low-pass filtering and power-line interference rejection.

#### 3.1.2. BLE Communication Performance

The data transmission rate of our system was calculated using the formula below: system sampling frequency × (data header length + (single channel data bit width) × number of channels) = 2000 Hz × (16 + (16) × 4 + 32) bit = 224,000 bit/s.

The low-power Bluetooth module we designed had a maximum baud rate of 921,600 bits, which fully met the system transmission rate requirements. To balance power consumption and transmission efficiency, the actual baud rate was set to 230,400 bits, and non-blocking transmission was achieved by setting the MCU and terminal’s serial port FIFO and verifying data integrity with parity checking. We used an oscilloscope (DS2202A, RIGOL Technologies Co., Ltd., Suzhou, China) to measure the average delay time of a single four-channel synchronous acquisition signal transmission, which was found to be 18 ms. The packet loss in three-minute collections at different transmitter-receiver distances was also measured using HC-T software (HC Technology, Guangzhou, China), to determine the maximum transmission distance to be 12 m (with a packet loss rate of less than 0.1%, outdoors).

#### 3.1.3. Current Consumption

During continuous operation of the system, we measured the power consumption of each component, as shown in Table 1. The wristband system was powered by an 800 mAh lithium-polymer battery and could continuously collect data for 4 h. The battery can be fully charged in 2.7 h (at a charging current of 300 mA). Low-power chips were used for the various components of the system to keep the overall power consumption within an acceptable range. Additionally, some units could be put in sleep/standby mode at appropriate time to significantly reduce current consumption (e.g., the MCU had a running current of 5.5 mA, a sleep current of 2.1 mA, the ADC chip had a running current of 20 mA, and a maximum standby current of 8 mA), thereby extending the working time.

#### 3.1.4. System Design Results

The main performance parameters of the system are presented in Table 2. Compared with other similar systems, our wristband system has the following advantages: (1) high signal sampling frequency and precision, wide signal bandwidth, and high signal gain; (2) more flexible wireless data transmission than wired data transmission; (3) the signal acquisition circuit placed on the wristband of the watch, which conforms to the wearing habit.

### 3.2. System Applications

#### 3.2.1. Fatigue Detection

Progress has been made in monitoring muscle fatigue using sEMG devices, and many researchers have used the time-domain feature (root mean square value, RMS) and frequency-domain feature (median frequency, MF) of sEMG signals to determine the degree of muscle fatigue. As fatigue deepens, RMS increases along with MF decreases, which has become a consensus in the field of muscle fatigue detection [32]. As follows, we have demonstrated through two experiments that the system can effectively detect the muscle fatigue.

(1) Dynamic contractions

**Figure 4:**
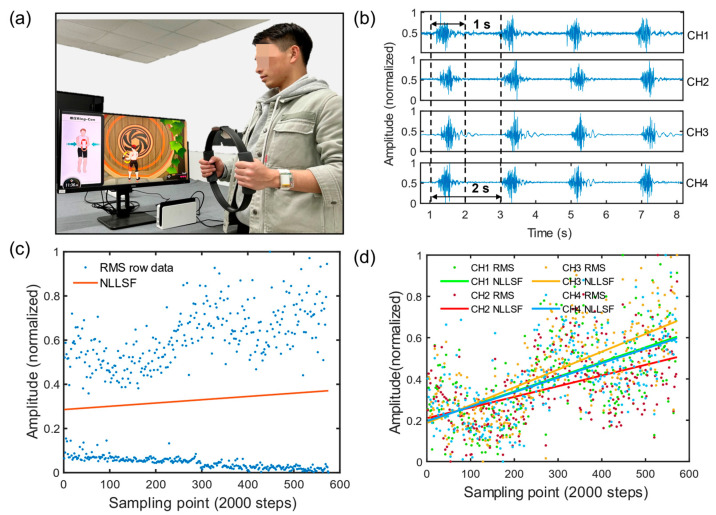
Intermittent force generation fatigue experiment. (**a**) Experimental method demonstrated with subjects periodically pressing the fitness ring. (**b**) Waveforms of sEMG signals generated by intermittent force generation acquired by the system. (**c**) System-acquired data RMS and the linear fitting curve of the upper boundary data points. (**d**) Four-channel RMS data curves with linear fit results after system acquisition data segmentation.

As shown in Figure 4a, subjects wore the system and performed intermittent force fatigue experiments using a fitness ring (Ring Fit Adventure, Nintendo Co., Ltd., Kyoto, Japan). A portion of the sEMG signal collected from four channels are shown in Figure 4b. The force cycle was 2 s with a force duration of 1 s, and the fitness ring needed to be compressed to its limit. The subjects compressed the fitness ring in a cycle until the fatigue limit. Figure 4c shows the processed data after the experiment, with the horizontal axis representing the sampling point sequence, and the vertical axis representing the RMS value calculated through 2000 sampling points with normalization. After processing the data with the least squares method, the data points were found to have obvious boundaries because the signal amplitude during the force period was significantly higher than during the non-force period. Therefore, the upper boundary data points were extracted and processed using the least squares method to obtain the fitting curve of the four-channel data. The experimental results showed that as the intermittent force progressed, the degree of muscle fatigue increased, and the wristband system could effectively detect the upward trend of the RMS of the four-channel myoelectric signals.

(2) Constant length (static) muscle contractions

**Figure 5:**
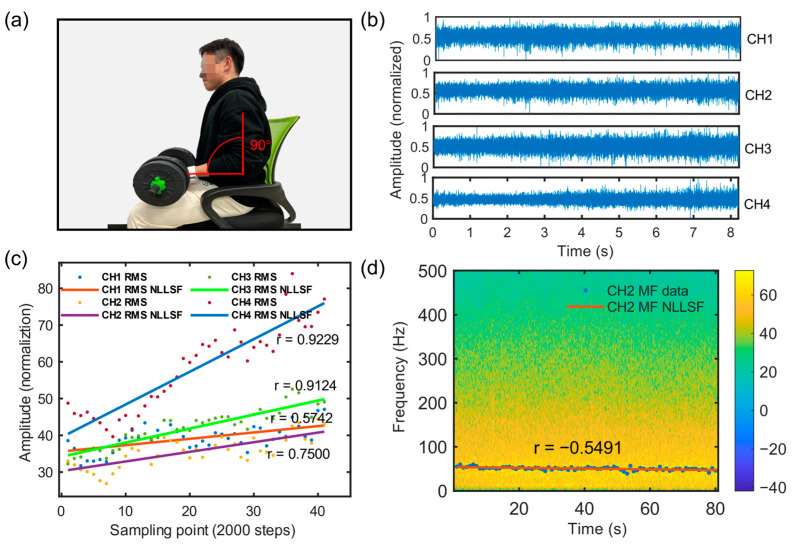
Continuous force fatigue experiment. (**a**) Experimental method demonstrated with the subject lifting a dumbbell at 90° of elbow flexion. (**b**) Waveforms of sEMG signals generated via intermittent force generation acquired by the system. (**c**) RMS curves of system-acquired data with their linear fitting results. (**d**) Time-frequency plot of system-acquired data, the median frequency curve, and linear fit results.

As shown in Figure 5a, subjects wore the EMG detection system and performed a sustained force fatigue test using a 10 kg dumbbell. The subject bent their elbow to 90 degrees, held the dumbbell, and maintained the position until exhaustion. The four-channel partial signals were collected as shown in Figure 5b, where higher signal amplitudes were observed during the force period compared to the non-force period in Figure 4b. Figure 5c shows the processed data, where the horizontal axis represents the sample point sequence, the vertical axis represents the RMS value calculated through 2000 sample points with normalization. Figure 5d shows the time-frequency graph of the sEMG signal after the short-time Fourier transform and is superimposed with median frequency data. As a result, the system can effectively detect the upward trend of RMS in the four-channel sEMG signals as muscle fatigue increases, as well as a gradual concentration towards the low-frequency area and a downward trend of the MF curve.

#### 3.2.2. Gesture Recognition

**Figure 6:**
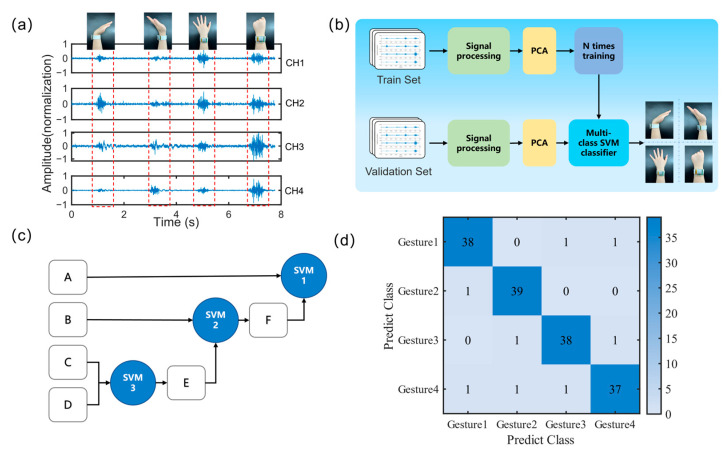
Four-category static gesture recognition experiment. (**a**) Experimental method and system acquisition data demonstration. (**b**) The flowchart of machine learning process. (**c**) The flowchart of multi-classification SVM algorithm. (**d**) The confusion matrix of experimental results.

In this study, machine learning algorithms were used to recognize four static hand gestures: fist clenching, hand opening, wrist flexion, and wrist extension, demonstrating the feasibility of the system in human–computer interaction applications, as shown in Figure 6a. In order to extract features from the sEMG signals, we used principal component analysis (PCA) to reduce the dimensionality of the data and improve its discriminative power. The reduced data were then input into a support vector machine (SVM) classifier. Compared to other classifiers such as multilayer perceptron (MLP), linear discriminant analysis (LDA), quadratic discriminant analysis (QDA), and decision tree (DT), SVM achieved higher classification accuracy on feature sets such as TD, AR4, and AR6 [33].

To collect training samples for the classifier, the four-channel sEMG signal acquisition system was used to acquire raw myoelectric signals. All experimental and computational tasks were performed on a workstation with hardware specifications of an Intel Core i9, NVIDIA RTX 3070, and 128 GB RAM, and the software environment of Windows 11 and MATLAB 2021a. The flowchart of the machine learning process is shown in Figure 6b, which includes both training and testing processes. In the training process, the electric signals generated by each hand gesture were combined into a matrix as the input for PCA for feature extraction, mainly to remove redundant information [34].

Subsequently, we classified the gesture patterns using a multi-class SVM algorithm (Figure 6c) with features extracted by PCA. Each class of the obtained gesture recognition patterns was set as a classifier sample, and a binary SVM classifier was trained using the remaining samples, followed by the creation of a multi-class classifier. The samples for the four categories of fist clenching, hand opening, wrist flexion, and wrist extension were labeled as A = 1, B = 2, C = 3, and D = 4, respectively. In the entire training process, the closest two classes (A and B) were firstly used to train SVM3. Then, these two classes were combined into one class named E. Afterwards, the distances between each remaining class were calculated, and SVM2 was trained using E and the closest class (C). Similar to the first step, the two classes for training SVM2 were combined into class C1. Finally, SVM1 was trained using the remaining two classes (C1 and B2). During the classification process, the binary tree was traversed from SVM1 to SVM3 step by step until the output of SVMi was positive.

The sEMG signal dataset used to train and test the model in this study was collected from 10 healthy subjects. Our proposed method achieved an average classification accuracy of 95% for four hand gestures, as shown in Figure 6d. The results of this research demonstrate the feasibility and practical value of sEMG gesture recognition based on a wrist muscle signal acquisition system in machine control (such as prosthetics and drones) and metaverse scenarios [35,36].

## 4. Conclusions

This study presents a soft electronic wristband with a four-channel sEMG signal electrode and signal conditioning circuit, as well as a core board circuit integrating control, ADC, wireless communication, and power management units. The performance of the system is evaluated using metrics such as frequency band range, wireless signal transmission performance, latency, and power consumption. A series of experimental results prove the accuracy and reliability of our wristband system for both muscle fatigue detection and gesture recognition applications.

In summary, our research provides a wearable sEMG detection system that combines advanced sensor technology, signal processing technology, and machine learning technology with a wearable wristband design. Compared with other studies, our system shows the following advances: (1) the system is worn on the wrist for human–computer interaction and fatigue detection, which is more in line with human wearing habits; (2) the signal acquisition circuit with wide bandwidth, high gain, high sampling rate, and sampling accuracy improves the quality of signal acquisition and provides accurate data for subsequent data analysis and algorithm processing; (3) the soft electronic wristband is assembled with flexible circuits and soft silicone package, making it more comfortable to wear while reducing the size of the area of core PCB.

This electronic wristband has the potential to replace the common wristband of commercial smartwatches by adding gesture recognition and muscle fatigue state detection functions based on the sEMG signals. In the future, we will continue to reduce the power consumption of the system, improve the signal acquisition quality, and deploy intelligent embedded algorithms, to achieve an ultra-low-power, portable, reliable and highly-integrated wristband system with signal acquisition, processing, and analysis capabilities.

## Figures and Tables

**Table 1:** The power consumption (unit: mW) of each component.

State	sEMG Signal Conditioner	ADC	MCU	BLE	Total
Working	33	100	66	23.1	222.1
Dormant	0	40	6.9	4	50.9

**Table 2:** Comparison of the main performance parameters between the system in this work and other similar systems.

Description	[1]	[29]	[30]	[31]	This Work
Number of channels	3	4	4	8	4
Site of collection	forearm	wrist	forearm	forearm	wrist
Bandwidth	34~398 Hz	20~450 Hz	9.57~511 Hz	60~450 Hz	15~500 Hz
Gain	922 V/V	500 V/V	1995 V/V	-	2492 V/V
A/D Resolution	14-bit	16-bit	12-bit	12-bit	16-bit
Sampling frequency	-	1 kHz	1.6 kHz	500 Hz	2 kHz
Communication	Wire	Bluetooth	Wi-Fi	Bluetooth	Bluetooth
Max. transmission distance	-	-	5 m	-	12 m
Power supply	Battery	Battery	Battery	Battery	Battery
Dimensions	-	15 mm × 44 mm	34 mm × 25 mm	85 mm × 50 mm × 6 mm	30 mm × 120 mm × 5 mm (Wristband) 45 mm × 36 mm × 12 mm (Core PCB)
